# Acute Respiratory Deterioration Following Parvovirus B19 Infection in a Patient With Interstitial Lung Disease: A Case Report

**DOI:** 10.1002/rcr2.70751

**Published:** 2026-09-01

**Authors:** Tomoki Kishaba, Satoshi Marumo, Yuya Nishida, Eri Nohara, Jukiya Higashi, Shohei Aoki, Chiaki Okura, Shiori Jinno, Noriyuki Tashima, Chie Morimoto, Takamasa Kitajima, Daiki Inoue

**Affiliations:** ^1^ Department of Respirology Kitano Hospital, The Tazuke Kofukai Medical Research Institute Osaka Japan

**Keywords:** acute exacerbation, interstitial lung disease, parvovirus B19, pulmonary fibrosis, viral infection

## Abstract

Acute exacerbation of interstitial lung disease (AE‐ILD) is often idiopathic, although viral infections are recognized precipitating factors. We report an adult patient with underlying ILD who developed acute respiratory deterioration in the setting of primary parvovirus B19 infection. A 70‐year‐old Japanese man was admitted to our department with a low‐grade fever and a facial rash. Two years prior to admission, the patient had been diagnosed with ILD, which had been treated with systemic corticosteroids that were subsequently discontinued following clinical improvement. High‐resolution computed tomography (HRCT) revealed newly developed bilateral ground‐glass opacities. Infection with parvovirus B19 was confirmed by serological testing and bronchoalveolar lavage results. Although the precise contribution of parvovirus B19‐associated lung injury, infection‐triggered AE‐ILD and acute exacerbation of the underlying ILD could not be definitively determined, steroid pulse therapy led to clinical and radiological improvement. This case highlights the importance of considering parvovirus B19 infection as a potential contributor to acute respiratory deterioration in patients with underlying ILD.

## Graphical abstract

A 70‐year‐old man with interstitial lung disease developed a fever and facial rash, with new bilateral ground‐glass opacities on high‐resolution computed tomography. Parvovirus B19 infection was confirmed, suggesting viral infection as a trigger of acute exacerbation through direct and immune‐mediated mechanisms.

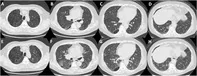

## 1 Introduction

Viral lower respiratory tract infections can lead to a spectrum of acute respiratory deterioration, most commonly manifesting as viral bronchitis and viral pneumonia. Furthermore, in patients with underlying interstitial lung disease (ILD), viral infections can precipitate an acute exacerbation of ILD (AE‐ILD). A prospective study detected respiratory viral infections in 19.2% of AE‐ILD cases, which were associated with poor short‐term outcomes [1].

Parvovirus B19 is a small, non‐enveloped single‐stranded DNA virus best known as the cause of erythema infectiosum in children. In adults, the infection is usually self‐limiting, occasionally accompanied by a rash or arthralgias and transmission occurs mainly via respiratory droplets. Beyond its typical manifestations, parvovirus B19 has been implicated in organ injury through direct viral and immune‐mediated mechanisms. Although the virus has been linked to chronic interstitial lung injury, parvovirus B19‐associated acute respiratory deterioration has rarely been reported in adults; herein, we describe a life‐threatening case.

## 2 Case Report

A 70‐year‐old Japanese man was admitted with low‐grade fever and a facial rash. Two years earlier, he had been diagnosed with ILD, suspected to be bird‐related chronic hypersensitivity pneumonitis (BRCHP) and treated with systemic corticosteroids, which were tapered and discontinued several months before admission. He was a former smoker (38 pack‐years). Following the initial suspicion of BRCHP, strict avoidance of avian antigens, including feather duvets and down jackets, was thoroughly instructed. One week before presentation, he developed a fever and rash after close contact with his daughter, who had upper respiratory symptoms. He was treated with oral levofloxacin without improvement and referred to our hospital.

On admission, he was alert, with a body temperature of 37.0°C and an oxygen saturation of 90% on room air, accompanied by bilateral fine crackles and a mild facial rash. C‐reactive protein level was mildly elevated (0.89 mg/dL), serum Krebs von den Lungen‐6 (KL‐6) and surfactant protein D (SP‐D) levels were unchanged from baseline, and the infectious workup was negative, including serum β‐d‐glucan, cytomegalovirus antigenemia, Aspergillus IgG and multiplex PCR for common respiratory pathogens. Given the recent sick contact and rash, parvovirus B19 serology was performed, confirming positive IgM and IgG.

**FIGURE 1:**
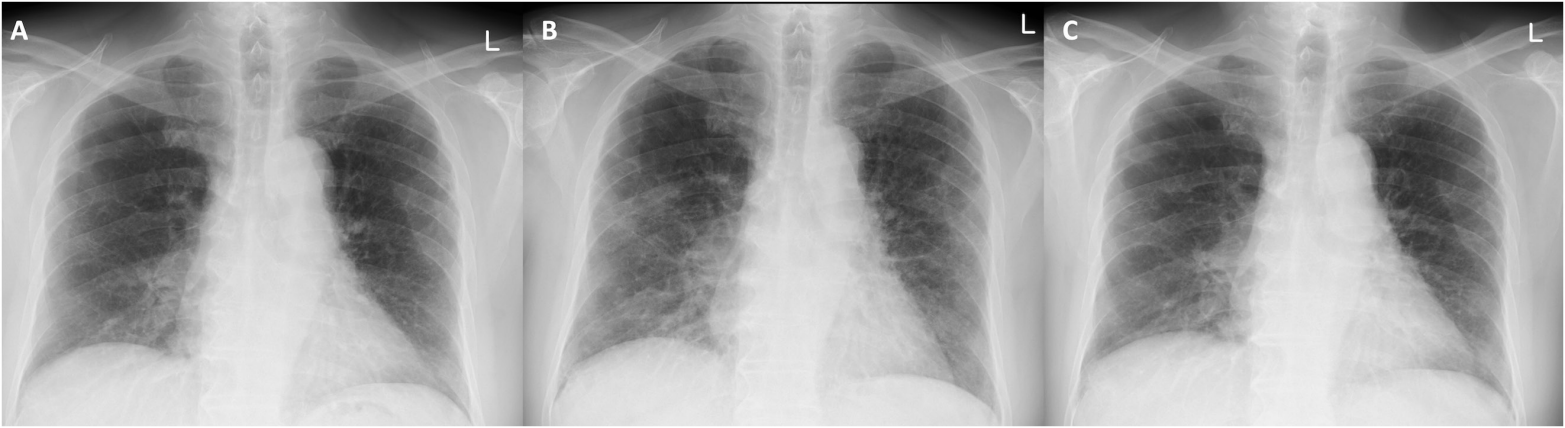
Serial chest radiographs. (A) Three months before presentation, normal findings. (B) At admission, decreased radiolucency in the bilateral middle and lower lung fields. (C) At 4‐month follow‐up, marked resolution of the opacities.

**FIGURE 2:**
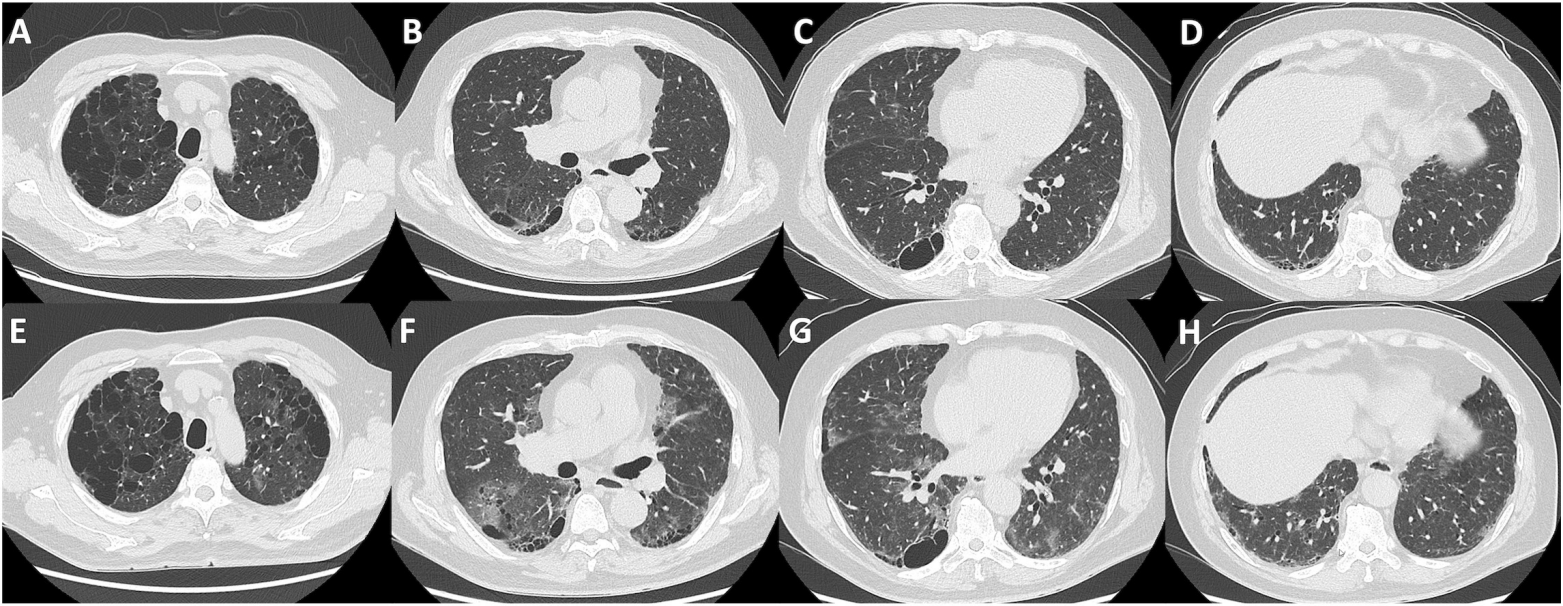
High‐resolution computed tomography (HRCT) images obtained 2 weeks before admission and at admission. (A–D) Scans obtained 2 weeks before admission showing no apparent new infiltrates. (D–H) Scans at admission demonstrating multiple newly developed bilateral ground‐glass opacities.

Chest radiography showed decreased radiolucency in the bilateral middle and lower lung fields (Figure 1A,B), and high‐resolution computed tomography demonstrated newly developed bilateral ground‐glass opacities (Figure 2A–H). Broad‐spectrum antibiotics were initiated for suspected bacterial pneumonia; however, the lung opacities progressed. Bronchoscopy was subsequently performed. Bronchoalveolar lavage (BAL) performed in the left B5 segment revealed an elevated total cell count of 1.42 × 10^5^ cells/mL, with a differential cell count showing lymphocytic predominance (lymphocytes, 63.5%; histiocytes, 33.8%; neutrophils, 1.0%; and eosinophils, 1.7%), and PCR testing of the lavage fluid was positive for parvovirus B19 DNA, while testing for *Pneumocystis jirovecii* was negative. No pathogenic organisms were isolated, and cytology was negative for malignancy. Based on these findings, acute respiratory deterioration associated with parvovirus B19 infection was considered.

During hospitalization, serum KL‐6 and SP‐D increased, and the oxygen demand rose transiently to 3 L/min. Steroid pulse therapy with methylprednisolone 1000 mg/day led to rapid clinical and radiological improvement. He was transitioned to tapering oral prednisolone and discharged on Day 19. At the 4‐month follow‐up, he remained stable with sustained radiological improvement (Figure 1C).

## 3 Discussion

The present report describes a patient with ILD, suspected to be BRCHP, who developed acute respiratory deterioration following parvovirus B19 infection microbiologically confirmed in the lung. Although the virus is a known cause of interstitial pneumonia or acute respiratory distress syndrome in children and immunocompromised patients, its clinical significance in adults with underlying ILD remains unclear. This case report describes a notable case of acute respiratory deterioration in a patient with underlying ILD, precipitated by a parvovirus B19 lower respiratory tract infection.

The clinical course of our patient was consistent with several possible mechanisms, including parvovirus B19‐associated lung injury, infection‐triggered AE‐ILD and progression of the underlying BRCHP. The diagnosis of AE‐ILD was considered based on rapid respiratory deterioration within 1 month and the appearance of new bilateral ground‐glass opacities on high‐resolution computed tomography, consistent with international consensus criteria [2]. However, distinguishing primary viral pneumonia from infection‐triggered AE‐ILD remains a clinical challenge due to the significant overlap in their clinical and radiological features [3].

Progression of the underlying BRCHP also cannot be completely excluded, as unrecognized antigen exposure may occur despite efforts to avoid avian antigen exposure. The BAL fluid findings further support this possibility. Although neutrophilic inflammation is commonly observed during acute exacerbation of ILD, the lymphocytosis observed in our patient may reflect progression of the underlying BRCHP. The favourable response to corticosteroids may also be compatible with the underlying BRCHP, which is generally considered more steroid‐responsive than progressive fibrotic ILDs such as idiopathic pulmonary fibrosis. However, these findings do not allow us to definitively distinguish between AE‐ILD and progression of the underlying BRCHP.

Consequently, although parvovirus B19 infection was confirmed, the extent to which it contributed to the acute respiratory deterioration and the relative contributions of viral lung injury, AE‐ILD and progression of the underlying BRCHP cannot be definitively determined.

The identification of parvovirus B19 as the causative trigger in the present case is supported by multiple findings. The patient had close contact with a family member with ‘slapped cheek’ syndrome, followed by facial erythema and fever. Serological testing showed elevated IgM and IgG titres, indicating acute primary infection. Most importantly, parvovirus B19 DNA was detected in bronchoalveolar lavage fluid during the acute phase. Although viral infections have been reported in approximately 19.2% of AE‐ILD cases, common pathogens such as influenza virus and herpesviruses were excluded. The absence of alternative infectious agents or heart failure further supports the possibility that parvovirus B19 infection contributed to the acute respiratory deterioration in this patient. Given the detection of parvovirus B19 in the lungs, we consider primary parvovirus B19 pneumonia or parvovirus B19‐induced AE‐ILD to be more plausible etiologies than an exacerbation of BRCHP. Consequently, the final diagnosis was established as acute respiratory deterioration precipitated by parvovirus B19 infection.

The pathophysiology by which parvovirus B19 triggers acute respiratory deterioration may involve both direct cytotoxic and indirect pro‐fibrotic mechanisms. Viral interaction with the P antigen on pulmonary endothelial cells may cause endothelial injury, increased vascular permeability and diffuse alveolar damage [4]. Experimental studies have shown that the viral non‐structural protein NS1 exacerbates lung fibrosis via TGF‐β/Smad pathway activation [5], while the VP1‐unique region induces inflammatory cytokine release and epithelial barrier disruption. In patients with pre‐existing fibrotic lung disease, these viral‐mediated processes may act as a ‘second hit’, precipitating acute exacerbation.

In conclusion, we report an adult patient with underlying ILD who developed acute respiratory deterioration in the setting of primary parvovirus B19 infection. Although the precise contribution of parvovirus B19‐associated lung injury, infection‐triggered AE‐ILD and acute exacerbation of the underlying BRCHP could not be definitively determined, this case highlights the importance of considering parvovirus B19 infection as a potential contributor to acute respiratory deterioration, particularly in patients with underlying ILD. Further studies are needed to clarify the mechanisms underlying parvovirus B19‐associated pulmonary involvement.

## Author Contributions

Tomoki Kishaba drafted the manuscript. Satoshi Marumo supervised the study and critically revised the manuscript. All authors reviewed and approved the final version of the manuscript.

## Funding

The authors have nothing to report.

## Disclosure

We did not use artificial intelligence (AI)‐assisted technologies in the preparation of this manuscript.

## Ethics Statement

This report was prepared in accordance with the ethical standards of our institution and the Declaration of Helsinki.

## Consent

The authors declare that written informed consent was obtained for the publication of this manuscript and accompanying images using the consent form provided by the Journal.

## Conflicts of Interest

The authors declare no conflicts of interest.

## Data Availability

The data that support the findings of this study are available on request from the corresponding author. The data are not publicly available due to privacy or ethical restrictions.
